# Supplementary material for: Multi‐dataset identification of innovative feature genes and molecular mechanisms in keratoconus
Source: J Cell Mol Med. 2024 Sep 19;28(18):e70079. doi: 10.1111/jcmm.70079 (PMC11412914; doi:10.1111/jcmm.70079)
Supplement: Supplementary file 2 — Material S2. [file JCMM-28-e70079-s002.zip › 7.GSEA analysis/5.RNF217-AS1/KEGG_COMPLEMENT_AND_COAGULATION_CASCADES.html]

Details for gene set KEGG\_COMPLEMENT\_AND\_COAGULATION\_CASCADES[GSEA]

|  || Dataset | RNF217-AS1.group.cls#h\_versus\_l |
| Phenotype | group.cls#h\_versus\_l |
| Upregulated in class | l |
| GeneSet | KEGG\_COMPLEMENT\_AND\_COAGULATION\_CASCADES |
| Enrichment Score (ES) | -0.4238046 |
| Normalized Enrichment Score (NES) | -1.3441516 |
| Nominal p-value | 0.060606062 |
| FDR q-value | 0.37843698 |
| FWER p-Value | 0.923 |
Table: GSEA Results Summary

  

Fig 1: Enrichment plot: KEGG\_COMPLEMENT\_AND\_COAGULATION\_CASCADES      
 Profile of the Running ES Score & Positions of GeneSet Members on the Rank Ordered List

  

| SYMBOL | TITLE | RANK IN GENE LIST | RANK METRIC SCORE | RUNNING ES | CORE ENRICHMENT || 1 | C4BPB | na | 705 | 0.487 | 0.0220 | No |
| 2 | F10 | na | 877 | 0.458 | 0.0570 | No |
| 3 | C5AR1 | na | 5106 | 0.213 | -0.0464 | No |
| 4 | MASP2 | na | 5547 | 0.198 | -0.0419 | No |
| 5 | C4BPA | na | 5574 | 0.197 | -0.0255 | No |
| 6 | BDKRB2 | na | 5730 | 0.192 | -0.0133 | No |
| 7 | C6 | na | 6523 | 0.167 | -0.0216 | No |
| 8 | KNG1 | na | 6976 | 0.154 | -0.0213 | No |
| 9 | C8A | na | 7260 | 0.145 | -0.0168 | No |
| 10 | C7 | na | 7568 | 0.138 | -0.0136 | No |
| 11 | C8G | na | 7627 | 0.137 | -0.0034 | No |
| 12 | CPB2 | na | 7806 | 0.132 | 0.0030 | No |
| 13 | CR1 | na | 8685 | 0.112 | -0.0125 | No |
| 14 | F7 | na | 9009 | 0.105 | -0.0127 | No |
| 15 | CD59 | na | 9539 | 0.094 | -0.0198 | No |
| 16 | FGB | na | 9642 | 0.093 | -0.0147 | No |
| 17 | TFPI | na | 9869 | 0.088 | -0.0135 | No |
| 18 | CD46 | na | 10049 | 0.086 | -0.0112 | No |
| 19 | C3AR1 | na | 10261 | 0.082 | -0.0101 | No |
| 20 | C9 | na | 10508 | 0.078 | -0.0105 | No |
| 21 | VWF | na | 11927 | 0.056 | -0.0465 | No |
| 22 | CD55 | na | 12780 | 0.045 | -0.0671 | No |
| 23 | F5 | na | 12959 | 0.043 | -0.0685 | No |
| 24 | PLAU | na | 13144 | 0.040 | -0.0704 | No |
| 25 | SERPINF2 | na | 13467 | 0.037 | -0.0765 | No |
| 26 | C1QC | na | 14192 | 0.028 | -0.0949 | No |
| 27 | MBL2 | na | 14280 | 0.027 | -0.0950 | No |
| 28 | THBD | na | 15201 | 0.017 | -0.1201 | No |
| 29 | C8B | na | 15799 | 0.011 | -0.1364 | No |
| 30 | FGG | na | 16254 | 0.006 | -0.1489 | No |
| 31 | CFD | na | 16262 | 0.006 | -0.1486 | No |
| 32 | BDKRB1 | na | 17163 | -0.003 | -0.1742 | No |
| 33 | C4A | na | 17397 | -0.005 | -0.1805 | No |
| 34 | PLG | na | 17835 | -0.009 | -0.1923 | No |
| 35 | F2 | na | 18355 | -0.014 | -0.2061 | No |
| 36 | F3 | na | 18449 | -0.015 | -0.2074 | No |
| 37 | PLAT | na | 19240 | -0.023 | -0.2283 | No |
| 38 | SERPINC1 | na | 19341 | -0.024 | -0.2291 | No |
| 39 | CFI | na | 19370 | -0.024 | -0.2278 | No |
| 40 | F12 | na | 20736 | -0.037 | -0.2640 | No |
| 41 | PLAUR | na | 20991 | -0.040 | -0.2678 | No |
| 42 | F8 | na | 22340 | -0.054 | -0.3020 | No |
| 43 | C4B | na | 23794 | -0.072 | -0.3376 | No |
| 44 | MASP1 | na | 23834 | -0.073 | -0.3324 | No |
| 45 | SERPINE1 | na | 24304 | -0.080 | -0.3390 | No |
| 46 | FGA | na | 24612 | -0.084 | -0.3405 | No |
| 47 | F9 | na | 25078 | -0.091 | -0.3460 | No |
| 48 | SERPINA5 | na | 27197 | -0.130 | -0.3957 | No |
| 49 | C1QA | na | 27255 | -0.131 | -0.3860 | No |
| 50 | F11 | na | 27563 | -0.138 | -0.3828 | No |
| 51 | KLKB1 | na | 28040 | -0.150 | -0.3835 | No |
| 52 | PROS1 | na | 28763 | -0.170 | -0.3896 | No |
| 53 | SERPING1 | na | 28908 | -0.174 | -0.3786 | No |
| 54 | F2R | na | 30147 | -0.211 | -0.3959 | No |
| 55 | F13A1 | na | 30597 | -0.227 | -0.3891 | No |
| 56 | A2M | na | 31800 | -0.279 | -0.3995 | Yes |
| 57 | CFB | na | 31827 | -0.281 | -0.3758 | Yes |
| 58 | F13B | na | 31989 | -0.291 | -0.3551 | Yes |
| 59 | C1R | na | 32064 | -0.295 | -0.3315 | Yes |
| 60 | CFH | na | 32351 | -0.313 | -0.3125 | Yes |
| 61 | PROC | na | 32402 | -0.317 | -0.2864 | Yes |
| 62 | C1QB | na | 32406 | -0.317 | -0.2588 | Yes |
| 63 | C5 | na | 32605 | -0.332 | -0.2357 | Yes |
| 64 | CR2 | na | 33316 | -0.387 | -0.2225 | Yes |
| 65 | C2 | na | 33778 | -0.443 | -0.1972 | Yes |
| 66 | C1S | na | 34217 | -0.532 | -0.1635 | Yes |
| 67 | SERPINA1 | na | 34257 | -0.544 | -0.1173 | Yes |
| 68 | SERPIND1 | na | 34543 | -0.680 | -0.0663 | Yes |
| 69 | C3 | na | 34660 | -0.822 | 0.0020 | Yes |
Table: GSEA details [plain text format]

  

Fig 2: KEGG\_COMPLEMENT\_AND\_COAGULATION\_CASCADES      
 Blue-Pink O' Gram in the Space of the Analyzed GeneSet

  

Fig 3: KEGG\_COMPLEMENT\_AND\_COAGULATION\_CASCADES: Random ES distribution      
 Gene set null distribution of ES for **KEGG\_COMPLEMENT\_AND\_COAGULATION\_CASCADES**
